# Genetic Heterogeneity in a Large Cohort of Indian Type 3 von Willebrand Disease Patients

**DOI:** 10.1371/journal.pone.0092575

**Published:** 2014-03-27

**Authors:** Priyanka Kasatkar, Shrimati Shetty, Kanjaksha Ghosh

**Affiliations:** Department of Haemostasis and Thrombosis, National Institute of Immunohaematology (ICMR), KEM Hospital, Parel, Mumbai, India; University of Bonn, Institut of experimental hematology and transfusion medicine, Germany

## Abstract

**Background:**

Though von Willebrand disease (VWD) is a common coagulation disorder, due to the complexity of the molecular analysis of von Willebrand factor gene (*VWF*), not many reports are available from this country. Large size of the gene, heterogeneous nature of mutations and presence of a highly homologous pseudogene region are the major impediments in the genetic diagnosis of VWD. The study is aimed at unravelling the molecular pathology in a large series of VWD patients from India using an effective strategy.

**Method:**

We evaluated 85 unrelated Indian type 3 VWD families to identify the molecular defects using a combination of techniques i.e. PCR-RFLP, direct DNA sequencing and multiple ligation probe amplification (MLPA).

**Results:**

Mutations could be characterized in 77 unrelated index cases (ICs). 59 different mutations i.e. nonsense 20 (33.9%), missense 13 (22%), splice site 4 (6.8%), gene conversions 6 (10.2%), insertions 2 (3.4%), duplication 1 (1.7%), small deletions 10 (17%) and large deletions 3 (5.1%) were identified, of which 34 were novel. Two common mutations i.e. p.R1779* and *p.L970del* were identified in our population with founder effect. Development of alloantibodies to VWF was seen in two patients, one with nonsense mutation (p.R2434*) and the other had a large deletion spanning exons 16–52.

**Conclusion:**

The molecular pathology of a large cohort of Indian VWD patients could be identified using a combination of techniques. A wide heterogeneity was observed in the nature of mutations in Indian VWD patients.

## Introduction

VWD is the commonest autosomal bleeding disorder caused by defects in VWF, a large multimeric, multifunctional glycoprotein involved in platelet adhesion and platelet aggregation and also in secondary hemostasis; it acts as a carrier for factor VIII (FVIII) [1]. VWD is classified into three distinct types; types 1 and 3 VWD result from quantitative deficiency in circulating plasma VWF while type 2 results from qualitative defects in VWF [2].

VWD is caused by defects in VWF gene (*VWF*), located in the short arm of chromosome 12 (12p13.3) spanning approximately 178 Kb, comprising of 52 exons. The mRNA is 8.7 kb in size, coding for precursor protein of 2813 amino acids that includes a 22 amino acids signal peptide, a propeptide of 741 amino acids and a mature subunit of 2050 amino acids [3]. A partial unprocessed pseudogene (*VWFP*) spanning exons 23–34 has been localized to chromosome 22q11.22-q11.23 with 97% homology to *VWF,* which makes it difficult to detect mutations in these regions [4].

VWD patients present with wide range of clinical manifestations varying from mild mucocutaneous bleeding like epistaxis, gum bleeding to life threatening haemorrhagic episodes like gastrointestinal and intracranial bleed. It is estimated to affect approximately 0.5–1.6% of the population in the Western countries [5]–[7]. Though there is no epidemiological data on the prevalence of different subtypes from India, type 3 patients outnumber the remaining subtypes mainly due to two factors i.e. high rate of consanguineous marriages in certain communities and the underdiagnosis of mild to moderate subtypes [8].

Worldwide, a wide heterogeneity in the nature of mutations has been reported in VWD patients (http://www.vwf.group.shef.ac.uk and https://grenada.lumc.nl/LOVD2/VWF). Though the mutations in *VWF* are distributed randomly throughout the gene, recurrent mutations have often been reported in VWD patients. Except a few small series reports on mutations in VWD patients, there is paucity of data on molecular pathology of type 3 VWD patients from India [9]–[12]. In one of the studies, it was also shown that around 28.5% of Indian VWD patients showed the presence of a common Arginine hotspot mutation i.e. p.R1659* observed in 6 out of 21 unrelated Indian patients [9]. Other types of mutations included missense mutations and deletions (9–33%), nonsense mutations (24–36%), splice site mutations (5–16%), insertion mutations (8–33%) and gene conversions (5–23%).

In the present study it was therefore planned to screen initially for the 11 CGA hotspot codons by the simple and inexpensive PCR-RFLP technique, followed by direct DNA sequencing. The aim was to elucidate the molecular pathology of a large series of VWD patients from India using a cost effective strategy and also to apply the data in the genetic diagnosis of the affected families

## Materials and Methods

### Ethics Approval

Ethics approval was granted by Institutional Ethics Committee for research on Human Subjects (National Institute of Immunohematology/Institutional Ethics Committee/28-2008).

A written informed consent was obtained from all the patients prior to the collection of blood samples. In case of pediatric patients, the written informed consent was obtained either from the parents or the caretakers.

### Patients

A total number of 85 unrelated severe VWD families (FVIII:C <10 IU/dL, VWF: Ag ≤ 5 IU/dL) attending the Comprehensive Hemophilia Care Center at Mumbai, India were included in the present study. These patients were referred from various Municipal and Private hospitals in Mumbai as well as from other parts of the country. Wherever available the parents, siblings, other affected and unaffected members along with key family members were also recruited for the study. Only in 30 families, the parents and/or other affected or unaffected members were available for molecular analysis. Since patients were referred from different parts of the country, the family members of the remaining patients were not available for the study. Clinical proforma was designed for obtaining the detailed clinical history along with the caste, sub caste and other details of all type 3 VWD patients. Bleeding history was derived from detailed questions on bleeding symptoms and a score was compiled to give the summed score results in a quantitative measure of bleeding severity [13]–[14]. Diagnosis of type 3 VWD was confirmed as per ISTH-SSC on VWF guidelines [2]. Family pedigree was obtained for each family extending to at least two generations.

### Phenotypic analysis

Venous blood samples were collected by phlebotomy in 3.2% sodium citrate (ratio of 9∶1 vol/vol) and EDTA. Plasma samples were assayed for VWF antigen levels (VWF:Ag) using commercial kits (Diagnostica Stago, Asnieres, France). Factor VIII coagulant activity (FVIII:C) was measured by one stage assay using semi automated coagulometer. Inhibitor against VWF was assayed by mixing studies of patients plasma and normal pooled plasma in various dilutions incubated at 37°C for one hour and then tested by i) aggregation with normal “O” group platelets using 1.25 mg/ml of ristocetin and ii) measurement of VWF:Ag levels by ELISA method [15].

### Strategy for the identification of VWF mutations

The strategy adopted for identification of mutations in VWF was to initially screen for the 11 CG-dinucleotide mutational hotspots that would result in a stop codon using PCR-RFLP technique. Those cases which were negative for these mutations were subjected to direct sequencing of all the 52 exons. Those cases wherein deletions were suspected were further confirmed by MLPA technique.

The genomic DNA was amplified for 11 CGA codons by PCR followed by restriction digestion with specific enzymes. The digested samples were run on 10% polyacrylamide gel electrophoresis. The bands were visualized by simple ethidium bromide staining using gel documentation system. All the PCRs were standardized in our laboratory and exclusion of pseudogene was confirmed. When no mutation was found, direct sequencing was carried out in the patients for all the exons of the *VWF*. Fifty five sets of primers for 52 exons were designed in house, using UCSC (http://genome.ucsc.edu), primer 3 output and the sequence from Ensembl. All the primers were obtained from Sigma Aldrich, Missouri, USA. Primers were designed so as to span the entire exon, intron-exon, as well as exon–intron boundaries and minimum 50–100 bases upstream or downstream to exonic region. The primers for known arginine hot spot regions were adapted from Baronciani *et al*, 2000. PCR for region of *VWF* corresponding to pseudogene region (exons 23–34) was standardized with stringent conditions to obtain *VWF* specific amplification [16]. Primer sequences, restriction enzymes are provided as supplementary files (**Table S1–S3 in File S1**).

### DNA extraction

Extraction of DNA from blood samples of patients and controls was done using commercial kits (Invitrogen, CA, USA).

### DNA sequencing

PCR conditions for 52 exons were standardized and directly sequenced using an automated DNA Sequencer (Applied Biosystems, Foster city, USA). Nucleotide numbering is as per Human Genome Variation society (HGVS) nomenclature (http://www.hgvs.org/mutnomen/).

### MLPA

Samples suspected to have deletions i.e. failure to get PCR amplification in consecutive exons, were further analyzed by MLPA technique (MRC-Holland, Amsterdam, The Netherlands). Fragment size analysis was performed using an ABI 3130 DNA sequencer. Data normalization was done using four healthy controls and calculation of copy number ratios for each exon specific amplicon was performed.

### In silico analysis

Any novel sequence variant, both silent and missense, was analyzed for their probable effect on VWF transcripts by using different prediction softwares i.e. PolyPhen-2 (http://genetics.bwh.harvard.edu/pph2/), SIFT (http://sift.jcvi.org/), Mupro (http://mupro.proteomics.ics.uci.edu) and Align GVGD (http://agvgd.iarc.fr). For the splice site mutations, the splicing potential score using four different prediction softwares were used i.e. HSF (http://www.umd.be/HSF/), Netgene2 (http://www.cbs.dtu.dk/services/NetGene2/), Maxentscan (http://genes.mit.edu/burgelab/maxent/) and Spliceport (http://spliceport.cbcb.umd.edu/).

### Haplotype analysis

Haplotyping using polymorphic markers of intron 40 VNTR was studied in ICs showing common mutations [17].

## Results

**Figure 1:**
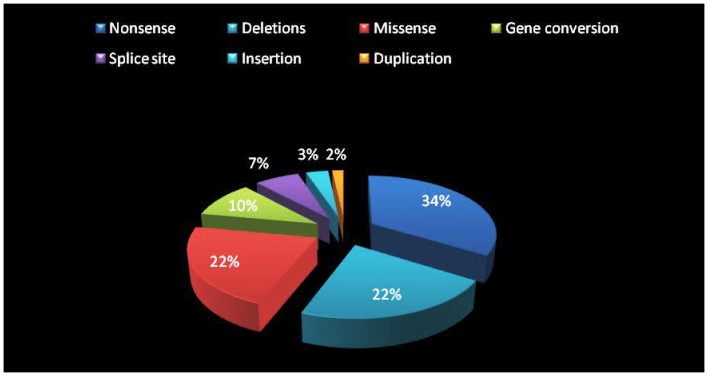
Different types of mutations identified in type 3 Indian VWD patients.

A total number of 85 unrelated type 3 VWD cases were studied. Mutations were detected in 77 (91%) unrelated ICs (**Figure 1**). Bleeding score in our patients varied from 2–34. The age of presentation of bleeding symptoms varied from 40 days to 25 years. 27 patients gave positive family history. 31 families gave history of consanguinity among parents. Two common mutations were identified in two communities in Indian population. Inhibitors to VWF were detected in two unrelated severe female congenital VWD patients.

**Figure 2:**
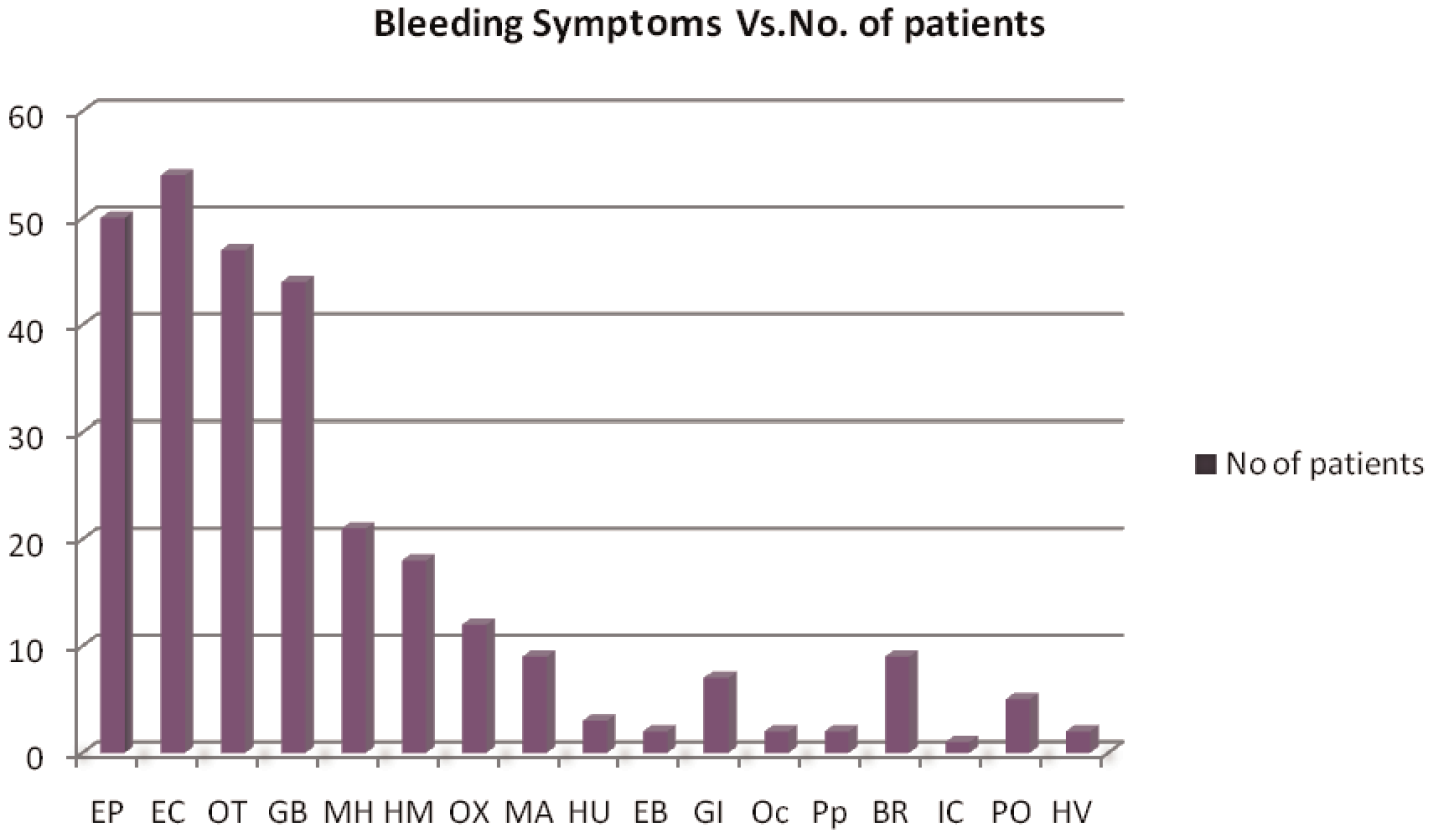
Graph of clinical manifestations seen in severe VWD patients. Epistaxis (EP), Ecchymosis (EC), On trauma (OT), Gum bleeding (GB), Menorrhagia (MH), Hemarthrosis (HM), On tooth extraction (OX), Malena (MA), Hematuria (HU), Ear bleed (EB),Gastro-intestinal bleeds (GI), On circumcision (OC), Post partum (PP), Bruising (BR), Intra cranial bleed (IC), Post operative (PO), Hematoma at the site of vaccination (HV).

A wide range of bleeding symptoms was seen in our patients which included epistaxis (50%), ecchymosis (54%), prolonged bleed after trivial trauma (47%), gum bleed (44%), menorrhagia (21%), hemarthrosis (18%), bleeding after tooth extraction (12%), bruising (9%), malena (9%), gastrointestinal bleed (7%), bleeding after circumcision (2%) and post partum hemorrhage (2%) (**Figure 2**). The bleeding score ranged between 2 and 34. As many patients were diagnosed early at the age of few months, the bleeding score was low in these patients. 13 out of 32 females in the reproductive age presented with a history of excessive bleeding during menses.

### Mutations

Mutations could be detected in 91% of the patients analyzed. 59 different mutations were identified in 77 unrelated ICs, 34 of which were novel – nonsense 20 (33.9%), missense 13 (22%), splice site 4 (6.8%), gene conversion 6 (10.2%), insertion 2 (3.4%), duplication 1 (1.7%), small deletion 10 (17%) and large deletion 3 (5.1%) (**Tables 1** **,** **2** **,** **3** **,** **4** **,** **5** **,** **6** **,** **7** **,** **8** **,** **9**). All the PCR protocols were standardized in the laboratory, including regions corresponding to pseudogene (exons 23–34).

**Table 1:** Nonsense mutations identified in type 3 VWD patients from India.

Pt ID	Age (years)	Sex	Exon	Nucleotide position	Aminoacid change	Zygosity	FVIII:C	VWF:Ag	Bleeding score	Family history	Consanguinity
1	10	M	3	c.100C>T	p.R34*	Hmz	<1	<1	11	-	+
2	32	F	8	c.970C>T	p.R324*	Hmz	1	<1	22	+	-
3	22	F	8	c.970C>T	p.R324*	Hmz	<1	<1	4	-	-
4	20	M	9	c.1093C>T	p.R365*	Hmz	<1	<1	22	+	+
5	49	F	9	c.1093C>T	p.R365*	Hmz	<1	<1	13	+	-
6	24	F	9	c.1093C>T	p.R365*	Hmz	1	<1	22	-	+
7	4	F	10	c.1117C>T	p.R373*	Hmz	<1	<1	24	-	+
8	2	F	10	c.1117C>T	p.R373*	Hmz	4.5	<1	11	-	-
9	2.5	M	28	c.4975C>T	p.R1659*	Hmz	7	1.5	9	-	-
10	42	M	31	c.5335C>T	p.R1779*	Htz	<1	<1	34	+	-
11	13	F	31	c.5335C>T	p.R1779*	Hmz	1	2.3	11	-	-
12	16	F	31	c.5335C>T	p.R1779*	Hmz	6	<1	16	+	-
13	3	M	31	c.5335C>T	p.R1779*	Hmz	5.4	<1	3	-	-
14	2	F	31	c.5335C>T	p.R1779*	Htz	3	2.6	6	+	+
15	29	M	31	c.5335C>T	p.R1779*	Hmz	1.8	<1	16	+	-
16	16	F	43	c.7300C>T	p.R2434*	Hmz	<1	<1	16	+	+
17	8	M	46	c.7300C>T	p.R2434*	Hmz	<1	1	7	-	+
18	8	M	47	c.7300C>T	p.R2434*	Hmz	8	4.6	8	-	+
19	1.3	F	45	c.7603C>T	p.R2535*	Htz	3.6	1	4	+	-
20	10	M	45	c.7603C>T	p.R2535*	Hmz	<1	<1	8	+	-
21	22	M	50	**c.8145C>A**	p.C2715*	Hmz	8	4.9	9	-	-
23	10	F	18	**c.2430C>A**	p.C810*	Htz	3.1	<1	8	+	+
24	0.8	F	14	**c.1693C>T**	p.Q565*	Hmz	2.4	2	3	-	+
25	26	M	14	**c.1693C>T**	p.Q565*	Hmz	2.5	<1	6	-	-
26	13	M	37	**c.6418C>T**	p.Q2140*	Hmz	<1	<1	8	+	-
27	16	F	45	**c.7651C>T**	p.Q2551*	Hmz	2	3.6	11	-	+
28	4	M	45	c.7558C>T	p.Q2520*	Hmz	3.2	3.2	7	+	-
29	0.8	F	45	c.7558C>T	p.Q2520*	Hmz	1.6	1.8	3	+	+
30	1.1	M	28	c.3931C>T	p.Q1311*	Hmz	<1	<1	4	-	+
31	16	F	8	**c.903T>A**	p.Y301*	C.	6.5	4	6	-	+
			22	**c.2878C>T, c.2880G>A**	p.R960*	Htz					
62	5	F	50	**c.8145C>A**	p.C2715*	Hmz	3.5	2.6	15	-	+
80	12	F	28	c.3931C>T	p.Q1311*	Hmz	3.2	<1	2	+	-
81	16	M	15	**c.1863C>A**	p.C621*	Hmz	2	4.2	6	-	-
82	2	M	15	c.1830C>A	p.Y610*	Hmz	1.4	<1	6	-	-
83	13	M	15	c.1812C>A	p.Y604*	Hmz	2	<1	6	-	-

Hmz: Homozygous, Htz: heterozygous, C.Htz: compound heterozygous, +: yes, -: no,

nomenclature according to the guidance issued by the Human Genome Variation Society (http://www.hgvs.org/) assessed on 13^th^ September 2013. Font in bold represents -novel mutation.

**Table 2:** Different deletions identified in Indian type 3 VWD patients.

Pt ID	Age (years)	Sex	Exon	Nucleotide position	Aminoacid change	Zygosity	FVIII:C	VWF:Ag	Bleeding score	Family history	Consanguinity
19	1.3	F	45	**c.7725_7729delCTGTG**	***p.R2575_E2577del***	Htz	3.6	1	4	+	-
32	18	M	22	**c.2908delC**	***p.L970del***	Hmz	3.2	<1	12	-	-
33	4.5	M	22	**c.2908delC**	***p.L970del***	Hmz	2.3	<1	10	-	-
34	21	M	22	**c.2908delC**	***p.L970del***	Hmz	2.2	<1	3	-	-
35	26	M	22	**c.2908delC**	***p.L970del***	Hmz	3	1	3	-	-
36	18	M	22	**c.2908delC**	***p.L970del***	Hmz	3	1	2	-	-
37	36	M	22	**c.2908delC**	***p.L970del***	Hmz	4.8	<1	8	-	-
38	21	F	22	**c.2908delC**	***p.L970del***	Hmz	4.8	<1	4	-	-
39	21	M	22	**c.2908delC**	***p.L970del***	Hmz	3.4	<1	2	-	-
40	8	M	22	**c.2908delC**	***p.L970del***	Hmz	3.8	2.7	4	-	-
42	13	F	22	**c.2908delC**	***p.L970del***	Hmz	<1	<1	8	-	-
43	11	M	22	**c.2908delC**	***p.L970del***	Hmz	5.2	3.4	4	-	-
44	59	M	22	**c.2908delC**	***p.L970del***	Hmz	2.8	<1	28	+	-
46	5	F	5	**c.497delA**	***p.N166del***	Htz	2.5	3.4	32	+	-
53	12	M	26	**c.3478delC**	***p.P1160del***	Hmz	2	<1	15	-	-
54	8	M	16	**c.2019_2020delTT**	***p.S673_Y674del***	Hmz	3.2	2	9	-	-
55	9	F	45	**c.7667_7679delTCTGCCCCTCGGG**	***p.V2556_G2560del***	Htz	3.2	<1	9	+	-
56	6	F	40	**c.6902_6909delCTCCCACG**	***p.A2301_T2303del***	Hmz	7	2.2	9	-	-
57	6	M	11	**c.1268_1270delTCT**	***p.F423del***	Htz	1.8	<1	12	+	-
59	16	M	45	**c.7568_7577delCCCCGGAGAA**	***p.S2523_N2526del***	Hmz	6	4	24	-	-
60	21	F	** Large deletion of exon 11-16**			Hmz	5.8	1	16	-	+
61	14	F	** Large deletion of exon 16-52**			Hmz	<1	<1	13	-	+
62	5	F	** Large deletion of exon 4-49**			Hmz	3.5	2.6	15	-	+
78	14	F	28	**c.4623_4624delGT**	***p.Q1541_Y1542del***	Htz	<1	<1	18	-	-

**Table 3:** Gene conversion identified in our patients.

Pt ID	Age (years)	Sex	Exon	Nucleotide position	Aminoacid change	Zygosity	FVIII:C	VWF:Ag	Bleeding score	Family history	Consanguinity
46	5	F	28	c.3686T>G	*p.V1229G*	Htz	2.5	3.4	32	+	-
				c.3692A>C	*p.N1231T*						
				c.3789G>A	*p.S1263S*						
				c.3797C>T	*p.P1266L*						
				c.3835G>A	*p.V1279I*						
				c.3862C>G	*p.L1288V*						
63	14	M	28	c.3931C>T	p.Q1311*	Hmz	7.5	<1	16	-	+
				c.4027A>G	*p.I1343V*						
				c.4079T>C	*p.V1360A*						
64	2	M	28	c.3835G>A	*p.V1279I*	Hmz	5.2	<1	6	-	+
				c.3931C>T	p.Q1311*						
65	10	M	28	c.3789G>A	*p.S1263S*	Hmz	2.1	1.4	5	-	-
				c.3797C>T	*p.P1266L*						
				c.3835G>A	*p.V1279I*						
				c.3931C>T	p.Q1311*						
				c.3951C>T	*p.A1317A*						
66	5	F	28	c.3862C>G	*p.L1288V*	Htz	1.4	2.3	6	-	-
				c.3931C>T	p.Q1311*						
				c.3951C>T	*p.A1317A*						
				c.4027A>G	*p.I1343V*						
67	13	M	28	c.3686T>G	*p.V1229G*	Hmz	<1	2.6	7	+	+
				c.3692A>C	*p.N1231T*						
				c.3931C>T	p.Q1311*						

**Table 4:** Missense mutations detected in type 3 VWD patients from India.

Pt ID	Age (years)	Sex	Exon	Nucleotide position	Aminoacid change	Zygosity	FVIII:C	VWF:Ag	Bleeding score	Family history	Consanguinity
10	42	M	20	c.2560C>T	*p.R854W*	Htz	<1	<1	34	+	-
14	2	F	28	**c.3862C>G**	***p.L1288V***	Htz	3	2.6	6	+	+
23	10	F	8	c.954T>A	*p.N318K*	Htz	3.1	<1	8	+	+
29	0.8	F	28	c.3835G>A	*p.V1279I*	Hmz	1.6	1.8	3	+	+
47	2	F	5	**c.478G>A**	***p.G160R***	Hmz	<1	2.3	6	-	+
48	2.5	M	18	c.2303G>A	*p.R768Q*	Hmz	2.5	<1	6	-	-
55	9	F	18	c.2303G>A	*p.R768Q*	Htz	3.2	<1	9	+	-
69	1.5	F	34	**c.5793G>C**	***p.Q1931H***	Hmz	<1	<1	2	-	-
72	6	F	17	**c.2208G>T**	***p.M736I***	Htz	2.5	4	4	-	-
78	14	F	28	c.4525A>G	*p.I1509V*	Htz	<1	<1	18	-	-
85	6	F	28	**c.4578G>T**	***p.Q1526H***	Htz	<1	2	2	-	+
87	16	F	28	**c.5001G>C**	***p.Q1667H***	Hmz	<1	5.3	12	-	+
			28	**c.5019G>C**	***p.E1673D***	Hmz					
			8	c.954T>A	*p.N318K*	Hmz					
89	16	F	28	c.4525A>G	*p.I1509V*	C.Htz	3.9	3	16	-	-
			8	c.954T>A	*p.N318K*						
97	10	F	45	**c.7604G>A**	***p.R2535Q***	Hmz	9	2.3	6	-	-

**Table 5:** Prediction software analysis of novel missense mutations detected in the present study.

Missense mutations	PolyPhen-2		SIFT		Align GVGD		MUpro	
	Confidence score	Prediction	Score	Protein function Prediction	Score	Prediction	SVM score	Protein structure stability
***p.M736I***	0.94	Possibly damaging	0.07	Affected	10.12	Less likely	-0.9109	Decreased
***p.L1288V***	0.997	Probably damaging	0	Affected	30.92	Likely	-0.8923	Decreased
***p.R2535Q***	0.057	Benign	0.04	Affected	42.81	Likely	-1	Decreased
***p.G160R***	1	Probably damaging	0	Affected	125.13	Most likely	-0.577	Decreased
***p.Q1526H***	0.277	Benign	0.04	Affected	24.08	Likely	-0.09	Decreased
***p.Q1931H***	0.02	Benign	0.04	Affected	24.08	Likely	-0.0114	Decreased
***p.Q1667H***	0.835	Possibly damaging	0	Affected	24.08	Likely	-0.592	Decreased
***p.E1673D***	0	Benign	0	Affected	44.6	Likely	-0.7235	Decreased

**Polyphen-2** - score 0 to1 shows low to high confidence for probability of protein damaging.

**SIFT**- mutations considered as pathogenic showing score <0.05.

**Align GVGD**- higher the score higher the probability of protein damaging nature of mutation

**MUpro** - higher the negativity in SVM score higher the probability of decrease in protein structure stability.

SVM- Support Vector Machine (SVM score – negative score for novel missense mutations showed the pathogenic probability).

**Table 6:** Insertions and duplication identified in Indian type 3 VWD patients.

Pt ID	Age (years)	Sex	Exon	Nucleotide position	Aminoacid change	Zygosity	FVIII:C	VWF:Ag	Bleeding score	Family history	Consanguinity
58	14	M	28	**c.3698_3699insAGT**	***p.L1232_C1234insKS***	Htz	5	3	9	-	+
71	7	F	28	**c.3698_3699insAGT**	***p.L1232_C1234insKS***	Hmz	2.5	<1	2	-	+
72	6	F	8	**c.901_902insCTA**	***p.E300_R302insSN***	Htz	2.5	4	4	-	-
68	6	M	37	**c.6487_6531dup**	***p.C2163_ I2177dup***	Hmz	1	<1	3	+	+

**Table 7:** Splice site mutation identified in Indian type 3 VWD patients.

Pt ID	Age (years)	Sex	Exon	Nucleotide position	Zygosity	FVIII:C	VWF:Ag	Bleeding score	Family history	Consanguinity
48	2.5	M	19	c.2443-1G>C	Hmz	2.5	<1	6	-	-
49	1.1	F	19	c.2443-1G>C	Hmz	4	2.6	3	-	-
50	12	M	19	c.2443-1G>C	Hmz	2	1	6	-	-
51	22	F	25	c.3379+1G>A	Htz	<1	4.8	9	-	-
70	22	M	16	**c.1946-1G>A**	Hmz	1.2	1	4	+	-
85	6	F	18	**c.2282-2A>G**	Htz	<1	2	2	-	+

**Table 8:** Prediction software analysis of splice site mutations detected in the present study.

Nucleotide change	Program	Score	
		Wild type	Mutated
c.2443-1G>C	HSF	82.26	71.24
	Netgene2	splice site normal	splice site destroyed
	Spliceport	splice site normal	splice site destroyed
	Maxentscan-MAXENT	8.16	0.09
	Maxentscan-MM	10.46	2.4
	Maxentscan-WMM	13.24	5.18
**c.1946-1G>A**	HSF	84.29	81.83
	Netgene2	splice site normal	splice site destroyed
	Spliceport	splice site normal	splice site destroyed
	Maxentscan-MAXENT	11.91	3.16
	Maxentscan-MM	12.5	3.75
	Maxentscan-WMM	14.79	6.04
**c.2282-2A>G**	HSF	72.42	72.54
	Netgene2	splice site normal	splice site destroyed
	Spliceport	splice site normal	splice site destroyed
	Maxentscan-MAXENT	2.94	-5.01
	Maxentscan-MM	3.88	-4.07
	Maxentscan-WMM	4.36	-3.59
c.3379+1G>A	HSF	81.32	74.8
	Netgene2	splice site normal	splice site destroyed
	Spliceport	splice site normal	splice site destroyed
	Maxentscan-MAXENT	8.56	0.38
	Maxentscan-MM	7.16	-1.02
	Maxentscan-WMM	5.29	-2.89

HSF- Human splicing finder.

Different scoring models used in MAXENTSCAN:

MAXENT- maximum entropy model.

MM- first order markov model.

WMM- weight matrix model.

**Table 9:** Different types of mutations identified in type 3 Indian VWD patients.

Nature of mutation	Total Number	Different types
Nonsense	20	9- Arginine to stop codon
		5- Glutamine to stop codon
		3- Cysteine to stop codon
		3- Tyrosine to stop codon
Deletions	13	3- Large deletions
		3- Deletion of single base
		7- Deletion of more than 2 bases
Missense	13	-
Gene conversion	6	-
Splice site	4	-
Insertion	2	-
Duplication	1	-

#### Nonsense mutations

Twenty different nonsense mutations were identified in 35 unrelated ICs (**Table 1**). Previously reported 8 CGA codon hot spot mutations were found in 20 unrelated ICs i.e. p.R34*, p.R324*, p.R365*, p.R373*, p.R1659*, p.R1779*, p.R2434* and p.R2535*. Three different Cysteine to stop codon mutations were identified in 4 unrelated ICs i.e. p.C621*, p.C810* and p.C2715* due to C>A substitution corresponding to cysteine residue that becomes a stop codon. Five different glutamine to stop codon mutations were found in 8 unrelated ICs i.e. p.Q565*, p.Q1311*, p.Q2140*, p.Q2520*, p.Q2551* due to a transition C>T in the glutamine residue that results in termination codon. p.Q1311* in two ICs (ID 30 and ID 80) was the only mutation in exon 28 that did not speculate on gene conversion and was found in heterozygous state in parents. Two tyrosine to stop codon mutations i.e. p.Y610* and p.Y604* were detected in 2 ICs. One IC (ID 31) was a compound heterozygote for novel mutations p.Y301* in exon 8 and p.R960* in exon 22. The mutation p.R960* was quite interesting as Arginine was converted to stop codon by the presence of two heterozygous changes (c.2878C>T, c.2880G>A) in the same codon.

#### Deletions

Three homozygous single base deletions identified were *p.P1160del*, *p.L970del* and *p.N166del*. All these mutations were also detected in parents as heterozygous deletions. Other small deletions detected in 7 unrelated ICs were *p.R2575_E2577del*, *p.S673_Y674del, p.V2556_G2560del*, *p.A2301_T2303del*, *p.F423del, p.Q1541_Y1542del* and *p.S2523_N2526del*. Three large homozygous deletions were identified in 3 unrelated ICs by MLPA technique. First IC (ID 60) had a large deletion of exons 11–16 whereas, second IC (ID 61) showed a deletion of exons 16–52 (**Table 2**). Studies in family members (parents and siblings) of two ICs (IDs 60 and 61) did not show the presence of these deletions. The third IC (ID 62) showed a large homozygous deletion from exons 4–49; both the parents showed this deletion in heterozygous state.

#### Gene conversion

Gene conversion as cause of type 3 VWD was seen in six unrelated ICs (ID 46, ID 63 - ID 67). Four ICs showed a homozygous gene conversion which involves 95-248 bases of recombination between native gene and pseudogene. Five ICs with this recombination event also had the nonsense mutation c.3931C>T (p.Q1311*) with several missense mutations. The sixth case (ID 46) was a compound heterozygote for a deletion mutation i.e. *p.N166del* with gene conversion. The mutations as part of the gene conversion were p.V1229G, p.N1231T, p.S1263S, p.P1266L, p.V1279I, p.L1288V, p.Q1311*, p.A1317A, p.I1343V and *p.V1360A* (**Table 3**).

#### Missense mutations

Thirteen different missense changes i.e. *p.G160R*, *p.N318K, p.M736I, p.R768Q, p.R854W, p.L1288V, p.R2535Q, p.V1279I, p.Q1931H, p.Q1667H, p.E1673D, p.I1509V, p.Q1526H* were found in the present study. Pathogenic probability of missense mutations was analysed using different prediction programs (**Table 4** **and** **Table 5**).

#### Insertion mutations

Two insertion mutations were identified in 3 ICs (ID 58, ID 71- ID 72). Two ICs (ID 58, ID 71) showed insertion of 3 bases AGT at c.3698 in exon 28. One IC (ID 72) had heterozygous insertion mutation of 3 bases CTA at nucleotide c.901 in exon 8. Both these mutations are novel (**Table 6**).

#### Duplication mutation

A duplication mutation of 48 bases *p.C2163_I2177dup* in one IC (ID 68) in exon 37 could be detected in homozygous state which was also detected in his brother. Parents were heterozygous for the same.

#### Splice site mutations

Four splice site mutations in 6 unrelated ICs (ID 48- ID 51, ID 70, ID 85) could be identified (**Table 7** **and** **Table 8**). A homozygous nucleotide change c.2443-1G>C was found in three different ICs (ID48- ID 50) in the acceptor site of intron 19. The fourth patient (ID 51) showed a heterozygous intronic change c.3379+1G>A in intron 25. The fifth patient (ID 70) showed a novel splice site mutation in intron 16 (c.1946-1G>A). The last patient (ID 85) had a splice site mutation in intron 18 (c.2282-2A>G); however mRNA studies are required to elucidate the pathogenicity of this variation.

### Mutations in VWD patients with alloantibodies

Inhibitor development to VWF could be detected in two female patients (ID 16 and ID 61). In the first case (ID 61), the patient was strongly positive for inhibitors with 89% of inhibition as compared to control plasma. This patient was homozygous for a large deletion spanning exons 16–52. Second patient (ID 16) showed presence of mild inhibitors to VWF with 38% inhibition. This patient showed a nonsense mutation i.e. *p*.R2434* in homozygous state; same mutation was detected in her sister and 2 other unrelated ICs, but they were negative for inhibitors.

### Common mutations

Two common mutations were identified in the present study.

1. *p.L970del* – A homozygous deletion *p.L970del* (i.e.c.2908delC) was detected in 12 ICs from Gujrat (Kachi Modh Ahmedabadi Ghanchi community from Western India) in exon 22. Mutation segregation analysis in the extended family members in all these families suggested a clear segregation of the mutant allele with the affected member (homozygous condition) and normal allele with unaffected member. Haplotype analysis using the three intron 40 VNTR markers showed a common haplotype suggesting a ‘founder’ effect.

2. p.R1779* - This mutation was detected in 5 unrelated ICs (4 homozygous and 1 heterozygous) from Uttarpradesh (Gaderia community from Northern India). One IC (ID 10) was a compound heterozygote for p.R1779* and showed the second heterozygous mutation in exon 20 (*p.R854W*). Haplotype analysis showed a common haplotype suggesting a ‘founder’ effect.

## Discussion

The present report is the largest series study on molecular pathology in type 3 VWD patients reported till date.

Mucocutaneous bleeding like epistaxis, ecchymosis and gum bleed were the commonest clinical manifestations [18]. Among women in the reproductive age (21%), menorrhagia was the chief clinical indicator. Four patients gave history of frequent transfusions due to heavy bleed during menses, which was managed with tranexamic acid and oral contraceptive pills. Hemoglobin (Hb) levels varied between the two sexes (males 8–16.5 g/dL, females 2.3–14.3 g/dL). Consanguineous marriage is a common practice in India; 31% type 3 VWD patients were born of consanguineous marriages. In the present study, 13 patients (6 males and 7 females) were identified with BS less than 4. Out of which 8 patients were aged less than 10 years. As patients were diagnosed early at the age of few months or years, they have fewer or no exposure to bleeding challenges such as tooth extractions, surgeries, menorrhagia etc. and score can be low in those pediatric ICs. Hence, the severity of the disease in paediatric patients is not conclusive when compared to adult ICs with the same mutations [18].

### Mutations in VWF

Genetic mutations responsible for type 3 VWD were found to be very heterogeneous, scattered throughout the *VWF*. In the present study, mutations could be characterized in 77 unrelated type 3 VWD patients using different methods i.e. PCR-RFLP, direct sequencing and MLPA technique. One of the major problems in mutation characterization in VWD patients is the co-amplification of pseudogene, the sequences of which often mimic the mutations in the authentic gene [16]. Stringent PCR conditions need to be applied during amplification of exons 23-34 for the selective amplification of *VWF*.

#### Arginine hot spot mutations

In the present study, nonsense mutations at arginine residues as a cause of type 3 VWD was found in twenty patients in 8 CGA codons. The IC (ID 3) homozygous for the p.R324* mutation with severe phenotype presented with mild bleeding tendencies, whereas, the same mutation was also identified in another patient with severe bleeding history since childhood. This mutation has earlier been reported in Indian and German patients [9], [19].

Previously reported *p*.R365* mutation [10] was identified in 3 unrelated patients. Patient ID 4 was born of consanguineous marriage with history of increased bleeding tendency, ecchymosis, gum bleed and hemarthrosis with FVIII:C and VWF:Ag levels of <1 IU/dL. The mutation was also seen in his sister who had history of gum bleed and menorrhagia. Patient ID 5 was diagnosed at the age of 49 years when she was referred to this Institute for prolonged bleed after tooth extraction. She had mild bleeding tendencies since childhood but gave history of post partum bleeding and death of her brother due to IC bleed. Her phenotypic assays showed severe FVIII and VWF deficiency. The same mutation was also identified in the remaining two siblings. This mutation has been reported in French as well as in Iran and Italian patients with severe VWD [10], [21].

*p*.R1659* has been reported as a common mutation (6/21) in Indian (North India) population by Gupta et al. earlier [9], However we could get in only 1 IC and a second study involving 14 Indian type 3 VWD patients (South India), did not show the presence of this mutation in their series [11]. The mutation has also been earlier reported by several other authors [10], [20].

A high prevalence of Arginine hot spot mutations were detected in the present study i.e. 23.5% of severe VWD patients which were detected by a simple PCR-RFLP technique. Similar results i.e. approximately 4–26% has been reported earlier in different populations [9], [11], [19], [20], [22], [23]. All these mutations are distributed throughout *VWF* (propeptide region, carboxyl terminus and most of the functional domains in mature *VWF*). It also gives an indication that approximately 23.5% of the mutations in Indian type 3 VWD patients could be characterized using a simple PCR-RFLP technique.

p.Q1311* was previously reported by several groups; most of the occurrences were with multiple mutations representing gene conversion [9], [11], [23]–[25]. This mutation was described by Casana *et al*, as common mutation in only four Spanish patients of Gypsy origin with similar haplotypes. The original Gypsy population is known to have migrated from north-west India and was settled in Europe i.e. Denmark to Spain [26]. The mutation p.R960* was very interesting as Arginine was converted to stop codon due to presence of two heterozygous changes in that codon.

#### Deletions

Novel deletions could be detected in 22% of our type 3 VWD patients resulting into a change in the reading frame and premature translational stop codon. Large deletions due to Alu mediated recombination have been reported in Chinese and Hungarian patients [27]–[28] as a result of homologous recombination event between Alu Y and Alu SP repetitive sequences. The presence of Alu sequence or chi sequences in intron/exon 16 junction could be predicted as a regional hot spot for genetic recombination in ICs (ID 60, ID 61). The pathological consequence of an additional nonsense mutation in exon 50 (p.C2715*) of patient (ID 62) is however not clear.

Three large deletions were identified in three patients (ID 60–62) with severe bleeding manifestations since childhood. No PCR amplification was seen in consecutive exons in the ICs which indicated the presence of large deletion of exons and this was subsequently confirmed by MLPA technique. Severe VWD due to large deletions has been reported previously by several workers [11], [27]. All three patients, where large deletions were found were born of consanguineous marriage and thus a heterozygous deletion would have been expected in parents. However PCR amplification of these exons showed normal sequences and MLPA analysis also did not show any difference in gene dosage as compared to reference samples in two families (ID 60, ID 61), whereas in the third family (ID 62), the parents were found to be heterozygous carriers of the deletion found in the index case. Thus, it appears that in the first 2 cases, there is a de novo deletion in the index case most probably as a result of recombination event due to repetitive Alu sequences in *VWF*. Deletion mapping however could not be done in these cases. Alu sequences are known to be hot spot for recombination events due to their transposition activity. A recent report by Ahmad et al [12] has also shown the break points of two large deletions in *VWF* in Indian type 3 VWD patients. The cause of these large deletions was found to be DNA double strand breaks followed by non-homologous recombination. Whether, non homologous recombination is the frequent cause of large deletions in Indian VWD patients needs to be investigated further.

#### Missense mutations

ID 47 showed a missense mutation i.e. *p.G160R* corresponding to the propeptide region. This region is important for VWF multimerization and intracellular transport, thus resulting in severe VWF deficiency. The missense change *p.L1288V* (ID 14) in A1-Domain (GpIb binding) results in decrease in the stability of the molecule, thus affecting its function. IC (ID 85) was a double heterozygote for *p.Q1526H* and a possible splice site mutation in D' domain (c.2282-2A>G) which plays important role in FVIII binding as well as multimerization of VWF. *p.I1509V* (ID 78 and ID 89) is a reported mutation, mutant residue had shortened side chain that lead to loss of two van der Waals interactions with Asn1610and Glu1504 [29].

Arginine at *p.R2535* (ID 97) is an important residue; substitution of glutamine in this region may potentially affect dimerization of VWF. Missense change *p.R768Q* (ID 48, ID 55) in the FVIII binding domain was identified which presumably affects the protein synthesis.

#### Gene conversion

Gene conversion is a fairly common occurrence reported earlier in 3 unrelated families (>10%) from India [24]. In our cohort gene conversion as recombinational event was identified in 6 unrelated ICs. There are two consensus chi sequences i.e. intron 27 and exon 28 in the VWF which is subject to gene conversion as a result of homologous recombination. It is also reported as rare events restricted to limited region of coding sequence i.e. D3 and A1 domain.

#### Splice site mutations

c.1946-1G>A mutation is located in the acceptor splice site of intron 15 that may affect the splicing in the propeptide region (domain A2) involved in multimerization and synthesis of VWF. However, a nucleotide change c.2443-1G>C in the acceptor splice site affecting the guanine was identified previously in Iranian patient [30]. The consequence of this change by prediction softwares is that it is likely to result in skipping of exon 19. Another splice site mutation c.3379+1G>A is located in the donor splice site of intron 25 which may result in skipping of exon 25. The possible splice site mutation in D' domain (c.2282-2A>G) may alter splicing, but needs further mRNA studies to elucidate the effect.

### Inhibitors in Inherited VWD

Development of Inhibitor to VWF is a rare phenomenon. Both the ICs (ID 61 and ID 16) had severe bleeding tendencies characterized by frequent episodes of mucocutaneous bleeds and menorrhagia. They had history of multiple transfusions with whole blood, cryoprecipitate or intermediate purity factor concentrates. On enquiry, both gave history of not responding to transfusion on recent hospital admissions.

Circulating alloantibodies directed against VWF develop in approximately 7–14% of type 3 VWD patients who have received multiple transfusions, yet there are only a handful of case reports in world literature. In most of these reports, antibodies have been found to be associated with large deletions in *VWF* [10], [27]. Except two reports, there is no other report of non deletional mutations associated with type 3 VWD in literature [10], [25]. Though there were three patients in this series with the mutation p.R2434*, they were negative for inhibitor. One of the reasons could be that they were minimally transfused. Number of exposures, other genetic predisposing factors or the environmental factors are also the triggering factors in eliciting an immune response in a patient. Baronciani et al, have described the presence of this mutation but the patient was negative for inhibitors [11].

Thus, we report high prevalence of Arginine hot spot mutations i.e. in 23.5% of severe VWD patients. RFLP is a cost effective and easy technique to detect mutations which can be applied in at least one fifth of our patients. All the stop codon mutations identified are distributed throughout VWF. It is likely that most of these mutations usually disrupt the synthesis of the VWF completely and result in almost undetectable levels of plasma VWF.

In 8 patients, no mutations could be identified despite sequencing all the exons of *VWF*. This is comparable to several other reports of mutation characterization in VWD patients, wherein different sensitivities (28–90%) have been reported using different mutation screening and direct DNA sequencing techniques [11], [31]–[33]. In two patients, second heterozygous mutation could not be identified, despite sequencing all the exons of *VWF* and MLPA analysis. Deep intronic mutations affecting mRNA splicing or creation of a cryptic splice site and introduction of additional in-frame exons are some of the plausible explanations for the disease manifestation.

The present study in a large cohort of Indian type 3 VWD patients adds further insight into the mechanism associated with VWD. The study also provides a basis for genetic diagnosis in all the affected VWD families, where the pathogenic mutations are identified.

#### Patient consent

Obtained.

## Supporting Information

File S1 **Tables S1–S3.** Table S1: Primer sequence for screening known Arginine hot spot regions of *VWF.* Blue coloured font indicates artificial restriction site has been introduced by using a mismatched nucleotide. The bold font in the oligonucleotides represents a mismatched nucleotide. Table S2: Mutation detection by PCR-RFLP for Arginine hot spot mutations. R- arginine, *- stop codon. Adapted from Baronciani et al., 2000. Table S3: Primers used for sequencing are shown in table.
(DOC)
