# Supplementary material for: Genetic Heterogeneity in a Large Cohort of Indian Type 3 von Willebrand Disease Patients
Source: PLoS One. 2014 Mar 27;9(3):e92575. doi: 10.1371/journal.pone.0092575 (PMC3967998; doi:10.1371/journal.pone.0092575)
Supplement: File S1 — Tables S1–S3. Table S1: Primer sequence for screening known Arginine hot spot regions of VWF. Blue coloured font indicates artificial restriction site has been introduced by using a mismatched nucleotide. The bold font in the oligonucleotides represents a mismatched nucleotide. Table S2: Mutation detection by PCR-RFLP for Arginine hot spot mutations. R- arginine, *- stop codon. Adapted from Baronciani et al., 2000. Table S3: Primers used for sequencing are shown in table. (DOC) [file pone.0092575.s001.doc]

**Table S1: Primer sequence for screening known Arginine hot spot regions of *VWF***

| OLIGO NAME | 5'<--------------SEQUENCE-------------->3' | LENGTH |
| --- | --- | --- |
| SauI Ex 3F | CCC TGA GAT CAC CAG CCC AA | 20 mer |
| SauI Ex 3R | AGC CCT CCC TCT GAA GTC CT | 20 mer |
| HhaI Ex 8F | ACA TCA ATG AAA TGT GTC AGG **C**G | 23 mer |
| HhaI Ex 8R | TGC TGG CAA GGT CTC TGA TCT | 21 mer |
| TaqI Ex 9F | ATG AGT GAC GCA ATG ACA ATG TT | 23 mer |
| TaqI Ex 9R | CCA GGC AGG TCT CCC AGA GCA | 21 mer |
| AluI Ex 10F | TTT TTC CCT CCA ACA GCA TT**A** GC | 23 mer |
| AluI Ex 10R | GGA GAC GCC TCC CCG ATT C | 19 mer |
| BsiEI Ex 28F | CAG CAG GCT ACT GGA CCT GG | 20 mer |
| BsiEI Ex 28R | GGG CGG TCG ATC TTG CTG AA | 20 mer |
| HhaI Ex 28F | GGA CAT CCT GCA GCG GG**C** G | 19 mer |
| HhaI Ex 28R | CCA GGC AGC CTC TTG ATC TC | 20 mer |
| DdeI Ex 28F | CCT GGT CTA CAT GGT CAC CG | 20 mer |
| DdeI Ex 28R | TAT CTT GGC AGA TGC ATG TAG C | 22 mer |
| HhaI Ex 31F | GAT GCC TTG GGC TTT GCT G**C**G | 21 mer |
| HhaI Ex 31R | AAC ATC CAA AAG TAA CCC CAG C | 22 mer |
| BsrI Ex 32F | GTC AGG CCA GTC CAT TTT GAG | 21 mer |
| BsrI Ex 32R | ATT TTT GTT TCT TTG GCG GGT TTA | 24 mer |
| HphI Ex 43F | TAT GTC TAC AGG TGT GTG T**T**C AC | 23 mer |
| HphI Ex 43R | CCT CTA CTT TCC CGC TCT GAT | 21 mer |
| DdeI Ex 45F | CTG CCC CAG CTT GTT GGA CT | 20 mer |
| DdeI Ex 45R | GCT TAA AGG TGG TGC CCG GT | 20 mer |

Blue coloured font indicates artificial restriction site has been introduced by using a mismatched nucleotide. The bold font in the oligonucleotides represents a mismatched nucleotide.

**Table S2: Mutation detection by PCR-RFLP for Arginine hot spot mutations**

| **Exons** | **Restriction enzyme used** | **Nucleotide substitution** | **Amino acid substitution** | **PCR size (bp)** | **Size of mutated fragments (bp)** | **Size of normal fragments (bp)** |
| --- | --- | --- | --- | --- | --- | --- |
|
| 3 | Sau96I | c.100C>T | *p.R34** | 247 | 40,207 | 40,40,167 |
| 8 | Hha1 | c.970C>T | *p.R324** | 97 | 97 | 23,74 |
| 9 | Taq1 | c.1093C>T | *p.R365** | 207 | 207 | 59,148 |
| 10 | Alu1 | c.1117C>T | *p.R373** | 119 | 22,97 | 119 |
| 28 | BsiE1 | c.4006C>T | *p.R1336** | 309 | 9,300 | 9,110,190 |
| 28 | Hha1 | c.4696C>T | *p.R1566** | 186 | 64,122 | 19,64,103 |
| 28 | Dde1 | c.4975C>T | *p.R1659** | 329 | 161,168 | 329 |
| 31 | Hha1 | c.5335C>T | *p.R1779** | 234 | 234 | 21,213 |
| 32 | Bsr1 | c.5557C>T | *p.R1853** | 303 | 6,141,156 | 6, 297 |
| 43 | Hph1 | c.7300C>T | *p.R2434** | 215 | 55,160 | 12,55,148 |
| 45 | Dde1 | c.7603C>T | *p.R2535** | 267 | 14,77,85,91 | 14,77,176 |

Adapted from Baronciani et al., 2000

Legends used: R- arginine, *- stop codon

**Table S3**: Primers used for sequencing are shown in table

| OLIGO NAME | 5'<--------------SEQUENCE-------------->3' | LENGTH (mer) |  |
| --- | --- | --- | --- |
| VWF_Ex1F | CAAAGCTTTATCAGCTTGGAGG | 23 |  |
| VWF_Ex1R | CAGGAGCAGGGATCAGTCA | 19 |  |
| VWF_Ex2F | GCTACTGCATGCCCTGATAGT | 21 |  |
| VWF_Ex2R | AGACACACCTGCTGATTCCC | 20 |  |
| VWF_Ex3F | TACGAGGCCAGAGAGGTTTG | 20 |  |
| VWF_Ex3R | ACCTTTCCGCTCAGACACTG | 20 |  |
| VWF_Ex4F | TTCTGCTGAGAAAAGGTTACGT | 19 |  |
| VWF_Ex4R | GAACATTTGCTTCCATTCTCTG | 20 |  |
| VWF_Ex5F | GCACCATGTTCTGAACCCTAC | 21 |  |
| VWF_Ex5R | TCACAGTGAGGCTTGAATGC | 20 |  |
| VWF_Ex6F | GCAGACCTAGAATTTTCACCC | 20 |  |
| VWF_Ex6R | CTGCCTAGCCTCCAGAACTATA | 20 |  |
| VWF_Ex7F | CAGGGCTAAGTCTCAGTGCC | 20 |  |
| VWF_Ex7R | AAGGGACACCACCCAGGA | 18 |  |
| VWF_Ex8F | CTCAGGCTGTGGCATGG | 20 |  |
| VWF_Ex8R | AGCAAACACAAGTGGCCTTC | 20 |  |
| VWF_Ex9F | TGGGGATTCTATAGTTGTGGG | 22 |  |
| VWF_Ex9R | GTTCTTTTCCACCTGCCACC | 21 |  |
| VWF_Ex10F | AGCTCTAAATCCATTTGCATACC | 23 |  |
| VWF_Ex10R | CCCTCCCTGTCTGGTAAGAG | 20 |  |
| VWF_Ex11F | GCATTCCACCTTGGCC | 16 |  |
| VWF_Ex11R | CAGGGACTGCCCATTCAG | 18 |  |
| VWF_Ex12F | ATTAAGAGGGTCCTGGGCTG | 20 |  |
| VWF_Ex12R | GGTTGAGAAGGAGGGTGCTA | 20 |  |
| VWF_Ex13F | AAATACATCTGCCTGCCACC | 20 |  |
| VWF_Ex13R | TTCTACCCAGAGCACAAGGG | 20 |  |
| VWF_Ex14FN | GCTAAACAACTATGCCGC | 18 |  |
| VWF_Ex14RN | GAACGCACTGCACTAATGT | 19 |  |
| VWF_Ex15FN | AGCAGCACTGGGCTATTTC | 21 |  |
| VWF_Ex15RN | CTACGCCCTCTTTCCACAG | 20 |  |
| VWF_Ex16F | AGCTACAAGGGGTGGCAAG | 19 |  |
| VWF_Ex16R | TCCATGAAGTAAAGGACTTGGG | 22 |  |
| VWF_Ex17F | AGTGGGAGGTGAAGATGTGG | 20 |  |
| VWF_Ex17R | GACGGTGTCACCCAGCTC | 18 |  |
| VWF_Ex18F | GAAGCCCAGGTGAGAAGATG | 20 |  |
| VWF_Ex18R | CACCACCTCCATTGCTATCC | 20 |  |
| VWF_Ex19F | GGCTCAAGTCTCAGACAACACT | 22 |  |
| VWF_Ex19R | AAGTGCGGAAGGTCCTGT | 18 |  |
| OLIGO NAME | 5'<--------------SEQUENCE-------------->3' | LENGTH (mer) |  |
| VWF_Ex20F | CACCCAGAGTATTCTGTGTTCCT | 22 |  |
| VWF_Ex20R | AGTACTCCAGTAGAAACCAGACCC | 21 |  |
| VWF_Ex21F | ATGCCAATCTTCTGGTCTGG | 20 |  |
| VWF_Ex21R | GGCTGTGCGTTATTCCATTC | 20 |  |
| VWF_Ex22F | ACTGAAGACAGATGGGCAGG | 20 |  |
| VWF_Ex22R | GACCTACGATCAGGGAGCAG | 20 |  |
| VWF_Ex23F | CTCCATTGCCGCCAGGAAT | 19 |  |
| VWF_Ex23R | CCCATGACAATGGGGACAGA | 20 |  |
| VWF_Ex24F | GGTCTCTTGGATCCTTCTGG | 20 |  |
| VWF_Ex24R | CACTCTGTGTCCATACCACCA | 21 |  |
| VWF_Ex25F | CCCAGACTAAGAGCCAGAGTT | 21 |  |
| VWF_Ex25R | CCATCCAGTCCCTACTAACACT | 22 |  |
| VWF_Ex26F | ACATAGCAAGACCCCATCTG | 20 |  |
| VWF_Ex26R | GAGGCTGAGATGAAGCAAGA | 20 |  |
| VWF_Ex27F | AGTTAAAAATGAGGCTTCCTC | 21 |  |
| VWF_Ex27R | TTTACCCAAAACCTAGTCTCTAA | 23 |  |
| VWF_Ex28ANF | ACTTGGATGTGGAATGGTCTA | 21 |  |
| VWF_Ex28AR | CAGGGGCCTGCTTCTCG | 17 |  |
| VWF_Ex28BNF | GCCTCCACCAGCGAGAT | 17 |  |
| VWF_Ex28BR | GTGTTGGTCCTGTTGCCG | 18 |  |
| VWF_Ex28CNF | GGTTCTGGATGTGGCGTAC | 19 |  |
| VWF_Ex28CR | GTATCTTGGCAGATGCCGCCG | 19 |  |
| VWF_Ex29F | CTGGTGGCCATTGTCCCT | 18 |  |
| VWF_Ex29R | GCCTCTTCGTCACCTGCTG | 19 |  |
| VWF_Ex30F | TTTGTGGCTCTACTTGATTCAA | 22 |  |
| VWF_Ex30R | ACTGGCCGAGGTCACAC | 17 |  |
| VWF_Ex31F | TCCACCGTTAAGACAGGGTG | 20 |  |
| VWF_Ex31R | CATCCAAAAGTAACCCCAGC | 20 |  |
| VWF_Ex32F | CATCTTCCTCATAGGGCTGA | 21 |  |
| VWF_Ex32R | CCCTGGGGTCTCTTGAATAC | 22 |  |
| VWF_Ex33F | GTCCCTATGTCTCCACTGTTAA | 18 |  |
| VWF_Ex33R | GACCAAAGGAGGAAAAATTAAC | 22 |  |
| VWF_Ex34F | CTCCTTGCTGTGTAGGCCT | 20 |  |
| VWF_Ex34R | GCACTGTGCATGTGGTGA | 19 |  |
| VWF_Ex35F | AACTTGGTCACACCTGCCTG | 20 |  |
| VWF_Ex35R | CAACTAAAAGCAACTGCCACC | 21 |  |
| VWF_Ex36F | GTGATGAATGTGCAGGAACTC | 20 |  |
| VWF_Ex36R | AAGCATCCAAGAGCCTCAG | 19 |  |
| VWF_Ex37F | ATGGCCTGTCTCTGGCATC | 19 |  |
| VWF_Ex37R | AGCCAGATAAAGAATCTGGGG | 21 |  |
| OLIGO NAME | 5'<--------------SEQUENCE-------------->3' | LENGTH (mer) |  |
| VWF_Ex38F | CATGTTGAATCAGCTGTGCC | 20 |  |
| VWF_Ex38R | AGTTGGAAGAGGCCAATCAC | 20 |  |
| VWF_Ex39F | AGGGAGAGCTGACCTCTGTG | 20 |  |
| VWF_Ex39R | GGTGAAGATGGGTGGCTG | 18 |  |
| VWF_Ex40F | AATCCCTCTGAGGCTGTCC | 21 |  |
| VWF_Ex40R | GACACCTTTCAGCACCTTCA | 21 |  |
| VWF_Ex41F | GGAATGTAGCATCCCACTCAC | 19 |  |
| VWF_Ex41R | TCTTGGAAGAGGTCCCTGAG | 18 |  |
| VWF_Ex42F | CTTCTGAGTCTAAAGCTCCCTGG | 23 |  |
| VWF_Ex42R | AATGGATGGGTGGATAGAAGG | 21 |  |
| VWF_Ex43F | GCGGCTTCTGTGTAGTAGGTG | 21 |  |
| VWF_Ex43R | ACCCTTCCTAAGATGCCCTC | 20 |  |
| VWF_Ex44F | CAGTACGCAGGAGGCGTAG | 19 |  |
| VWF_Ex44R | GGCAGGGAATGAGATGAAAC | 20 |  |
| VWF_Ex45F | TCTAGAAACCACCTTCCTGAGAG | 23 |  |
| VWF_Ex45R | CAGGAGCCAAAAGTGGAAAG | 20 |  |
| VWF_Ex46F | CGACCGATACAGGAGGGAG | 19 |  |
| VWF_Ex46R | CTCTGGTTTCCTTCATTTCTGC | 22 |  |
| VWF_Ex47F | GAGTGAGGTGAGTGGGGG | 18 |  |
| VWF_Ex47R | AAGACCGCAGTGAGGTCC | 18 |  |
| VWF_Ex48F | TGAGAGGCCAGCAAAATCAG | 20 |  |
| VWF_Ex48R | TGAACCAAACTTAGCCCCTC | 20 |  |
| VWF_Ex49F | CAAGCTCATGGTTTGAAGTGG | 21 |  |
| VWF_Ex49R | GAGAAATTATGCCGGAGCTG | 20 |  |
| VWF_Ex50F | CCCGGAGTGACCTGAAAG | 20 |  |
| VWF_Ex50R | AATGGGTTTCAAGGAGCAAG | 20 |  |
| VWF_Ex51F | AGGGCACTGGGGCTGA | 16 |  |
| VWF_Ex51R | TGCGAATTTTCCAGATCCTTC | 21 |  |
| VWF_Ex52F | GGTCAGGGAGAAAGCAGGC | 19 |  |
| VWF_Ex52R | GCCTGTGCTTCCTTCCTCAG | 20 |  |
|  |  |  |  |

**Supporting information legends:**

**Table S1: Primer sequence for screening known Arginine hot spot regions of *VWF***

**Legends:** Blue coloured font indicates artificial restriction site has been introduced by using a mismatched nucleotide. The bold font in the oligonucleotides represents a mismatched nucleotide.

**Table S2: Mutation detection by PCR-RFLP for Arginine hot spot mutations**

**Legends**: R- arginine, *- stop codon

**Table S3: Primers used for sequencing are shown in table**
